# Combining semi-synthesis with plant and microbial biocatalysis: new frontiers in producing a chemical arsenal against cancer†

**DOI:** 10.1039/c8ra02184h

**Published:** 2018-06-11

**Authors:** Samuel Gary, Janet Adegboye, Brian Popp, Jean-Christophe Cocuron, Brooklyn Woodrum, Nik Kovinich

**Affiliations:** Undergraduate Intercollegiate Biochemistry Program, Division of Plant and Soil Sciences, West Virginia University 333 Evansdale Drive 26506 Morgantown USA nikovinich@mail.wvu.edu; Department of Molecular Genetics, Ohio State University Columbus Ohio USA; Eugene Bennett Department of Chemistry, West Virginia University Morgantown USA; Division of Plant and Soil Sciences, West Virginia University Morgantown USA

## Abstract

Natural products (NPs) that exhibit anticancer activities are frequently not potent enough to be used clinically as therapeutics. Semi-synthesis and metabolic engineering are promising approaches for producing more efficacious derivatives of anticancer NPs (ACNPs), but each technique alone can be inefficient at obtaining specific ACNP derivatives that may be suspected to have enhanced anticancer activity. Here, we demonstrate that the methods of semi-synthesis and biocatalysis can be used as modules in succession and in different combinations to produce 6,8-dibromogenkwanin, a derivative of the ACNP apigenin. Further, we demonstrated that soybean seed coats can be used as a biocatalyst to convert brominated flavonoids into multiple derivatives. A strength of the combinatorial (bio)synthesis approach was that the order of the modules could be rearranged to increase the yield of the desired product. At lower treatment concentration (5 μM), 6,8-dibromogenkwanin exhibited enhanced antiproliferative activities against HT-29 colorectal adenocarcinoma cancer cells under normoxic and hypoxic conditions compared to its ACNP precursors, but not at higher concentrations. Dose–response analyses suggested that dibromogenkwanin had a distinct mode-of-action compared to apigenin. Thus, this proof-of-concept paper demonstrates combinatorial (bio)synthesis as an approach that can be used to produce novel chemistries for anticancer research.

## Abstract 2

Combinatorial (bio)synthesis combines modules of semi-synthesis and biocatalysts to produce novel derivatives of anticancer natural products for bioactivity testing.

## Graphical abstract

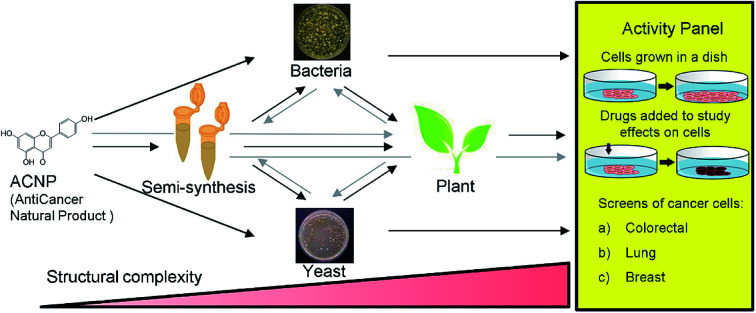

## Introduction

Bioactive natural products (NPs) commonly must be administered at concentrations that are too high to permit clinical use. High drug dosages generally increase the likelihood of off-target molecular interactions that can disrupt cellular processes and lead to adverse side effects. For this reason, drugs that bind single cellular targets at low micromolar or lesser concentrations are most suitable for use as drugs. The vast majority of small molecules that were approved by the U.S. FDA as anticancer drugs between 1981 and 2014 were derived from NPs and were mostly semisynthetic derivatives.^1^ The modification of NPs by semi-synthesis generally serves to improve drug efficacy by enhancing: (i) the strength of molecular interaction with the cellular target, (ii) the chemical stability, or (iii) the transport. Modification of the NP structure can also reduce the number of cellular targets and thus adverse side effects.

Semi-synthesis methods for chemical modification are generally highly efficient and amenable to scale-up. However, approaches can have limitations, particularly in maintaining stereochemistry or in making certain regiospecific modifications without the need for additional blocking agents that can be costly or environmentally unfriendly. With the advent of genetic engineering, biocatalysts such as engineered bacterial or yeast have emerged as important alternatives for drug modification. The benefits of biocatalysis are that enzymes generally maintain stereochemistry and are highly regiospecific. Yet, a shortcoming of using biocatalysis to modify NPs can be that enzymes are very limited in the substrates that they accept. For these reasons, novel methods are continuously being developed in metabolic engineering and semi-synthesis to circumvent the limitations to generate new drug molecules that have enhanced capability and efficiency. Yet, there has been little focus on combining the methods of biocatalysis and semi-synthesis to (bio)synthesize derivatives of NPs that are suspected to possess enhanced medicinal activities but cannot be efficiently produced by either method alone.

Flavonoids are a family of polyphenol secondary metabolites that have a 3-phenylchromen-4-one backbone. More than 6000 flavonoids are biosynthesized in plants. Epidemiological studies have associated flavonoids with diverse health benefits including reduced incidence of the onset of certain types of cancers. This has stimulated the testing of various flavonoids as antiproliferative and antitumor agents. Despite the notable anticancer activities of a broad spectrum of flavonoids, few molecules have exhibited strong enough potency to warrant their assessment as therapeutics in clinical trials. Exceptions include the soybean-derived isoflavone genistein, a highly specific inhibitor of protein tyrosine kinase (PTK) that blocks the mitogenic effect mediated by EGF.^2^ Genistein is currently the focus of Phase 2 clinical trials for the treatment of colorectal, bladder, brain and other types of cancers. Since the vast majority of flavonoids are not potent enough for clinical use, this has stimulated efforts to develop derivatives that have enhanced anticancer activities. A successful example is flavopiridol, a semi-synthetic derivative of rotukine that was approved by the Food and Drug Administration (FDA) to treat B-cell chronic lymphocytic leukemia (B-CLL) and acute myeloid leukemia (AML) and is in Phase I – II clinical trials for the treatment of a number of other cancers. Since so many flavonoid molecules have moderate antiproliferative and/or antitumor activities, a logical step in developing novel anticancer drugs may be to modify such molecules in ways that are suspected to enhance their bioactivities.

**Fig. 1:**
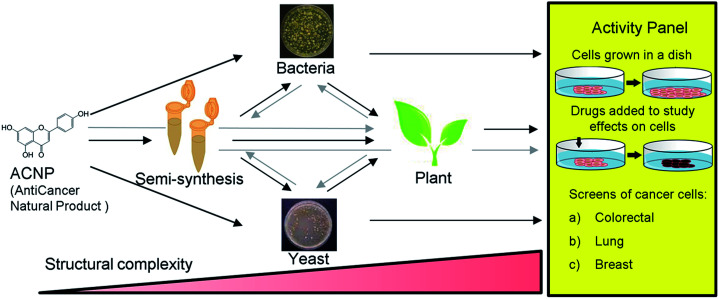
Schematic representation of the combinatorial (bio)synthesis approach for producing derivatives of anticancer natural products (ACNPs) for bioactivity testing. The process begins with an ACNP; mainly a plant phenylpropanoid or (iso)flavonoid. The ACNP may be modified by semi-synthesis prior to being fed to a bacteria, yeast, or plant cultures. Subsequent to a preliminary round of modification, the unnatural derivative may be further altered by feeding to plant cultures prior to being testing in the activity panel. Each successive round of modification increases the structural complexity of the compound. The activity panel consists of antiproliferation assays of human cancer cell lines.

Apigenin is a member of the flavone subclass of flavonoids that has notable anticancer activity but must be administered at relatively high concentrations (50–100 μM) to inhibit the proliferation of cancer cells by more than 50%.^3,4^ Apigenin induces apoptosis at these high treatment concentrations possibly by inhibiting a pathway that results in NF-κB activation.^3–5^ Due to its insufficient potency, apigenin is a prime candidate for the improvement of its anticancer activities by derivatization. Halogenation of the 6- and 8- positions of the phenylchromen ring is a modification that has been found to enhance the antioxidant activity and lipophilicity of several flavonoids, and render them more potent in inhibiting the proliferation of cancer cell lines compared to their precursor NPs.^6,7^ Further, methylation of specifically the 7-hydroxyl of apigenin to produce genkwanin significantly alters the anticancer activities.^8^ Based on these studies, we hypothesized that methylating the 7-position in addition to brominating the 6- and 8-positions of apigenin would enhance its anticancer activity. Since 6,8-dibromogenkwanin could not be produced using current semi-synthesis or biocatalysis protocols alone, we proposed a novel combinatorial approach. Beginning with the anticancer NP (ACNP) apigenin, we aimed to determine whether the methods of semi-synthesis and microbial biocatalysis could be performed in succession in different combinations to produce 6,8-dibromogenkwanin. Further, we demonstrated soybean seed coats as a plant biocatalyst for converting brominated flavonoids that did not exhibit enhanced anticancer activity into novel derivatives. The combinatorial (bio)synthesis approach can be viewed as a novel approach that takes advantage of the strengths of each method (*i.e.* the high yield of semi-synthesis, the regiospecificity of recombinant enzymes expressed in microbial systems, and the promiscuity of plant metabolism) to generate a plethora of structural variants with increasing structural complexity from an ACNP (Fig. 1).

## Materials and methods

### Materials

The rice (*Oryza sativa*) *OsNOMT* gene codon-optimized for expression in *E. coli* (GenBank accession MF991211) was synthesized by Invitrogen (Carlsbad, CA). Apigenin and genkwanin were from Carbosynth (San Diego, CA) and VWR (Radnor, PA), respectively. NBS was from Sigma Aldrich (St. Louis, MO). UPLC-MS solvents were Optima grade and flash chromatography solvents were from Fisher Scientific (Hampton, NH). NMR solvents were from Cambridge Isotope Laboratories (Tewksbury, MA). HT-29 and HCT 116 cells were generous gifts from Houjian Cai (University of Georgia).

### Biocatalysis with *E. coli* expressing synthetic *OsNOMT*

Synthetic *OsNOMT* was Gateway recombined into pDEST42 using the recombinase LR Clonase II (Thermofisher Scientific, Pittsburgh, PA). Fully sequenced constructs were transferred into *E. coli* BL21(DE3) pLysS (Novagen). For optimal biocatalysis of apigenin, cells were cultured in 30 mL of LB broth in 250 mL flasks to an OD600 of 0.8, then induced with 1 mM of IPTG. After 4 hours, apigenin or 6,8-dibromoapigenin was added to a final concentration of 60 μM from a 6 mM stock in DMSO. Following overnight culture, the bacterial pellet was extracted with 80% ethanol (v/v) by heating 80 °C for 3 hours. Ethanol was evaporated under a stream of nitrogen gas, water was added to 5 mL, and the aqueous phase was extracted three times with an equal volume of ethyl acetate. The bacteria growth medium was also extracted three times with an equal volume of ethyl acetate. Residues from bacteria pellet and growth medium were resuspended in ethanol and pooled for analysis by LC-MS or DMSO-d_6_ for analysis by NMR.

### Semi-synthesis

Bromination of flavonoids was performed as indicated.^9^ Briefly, *N*-bromosuccinimide (0.036 mmol) in 0.5 mL acetone was added dropwise to 0.018 mmol of flavonoid in 0.5 mL acetone. The mixture was stirred at 22 °C for 43 min, then extracted three times with an equal volume of ethyl acetate to remove succinimide. Reaction with apigenin yielded a bright yellow powder whereas genkwanin yielded a brown-orange amorphous solid. 6,8-Dibromo-genkwanin was purified from mono- and tri-brominated products by flash chromatography on silica gel 60 (particle size 0.040–0.063 mm, 230–400 mesh ASTM) from Silicycle (Ville de Québec, QC) using a gradient 10% → 40% ethyl acetate/hexanes with constant 5% methanol. Fractions were evaluated by TLC and like samples combined for analysis by ^1^H NMR spectroscopy.

### Biocatalysis with soybean seed coats

*Glycine max* (L.) Merr. line 107 was obtained from the Agriculture and Agri-Food Canada Soybean EMS-mutagenesis collection 2005 (M. Morrison, Eastern 140 Ontario Oilseed Research Centre, Ottawa). This line was selected due to its early maturation and its *I*^*i*^*RT* genotype. Plants were grown in an incubator as indicated.^10^ On ice, growing seeds (25–75 mg fresh weight) were isolated from pods and the seed coats were dissected from the embryos.^11^ Seed coats (8–10), hydrogen peroxide (1 mM final) (Sigma-Aldrich St. Louis, MO), and dibromoapigenin (1 mM final, from 100 mM stock dissolved in DMSO, 0.05% Tween20, 5% MeOH) were added to 970 μL of Milli-Q®-purified water in a 24-well microtiter plate. Plates were incubated in the dark for 24 hours at 22 °C on a rotary shaker (100 rpm). Seeds and reaction medium were then extracted for analysis of reaction products. Seeds were lyophilized to dryness then extracted with 80% ethanol (20 μL mg^−1^ dry weight) for 24 hours in the dark at 22 °C on a rotary shaker (100 rpm). Milli-Q®-purified water used to rinse the microtiter plates was pooled with the reaction medium. The mixture was extracted three times with equal volumes of ethyl acetate. Following evaporation to dryness under a stream of nitrogen gas, the residue was resuspended with seed extract. The combined seed and reaction medium extract was then filtered through 0.2 μm for analysis by LC-MS^n^.

### Chemical analyses

Compounds were structurally characterized by exact mass UPLC-PDA-MS^n^ and by ^1^H NMR spectroscopy. Reaction extracts were evaporated under a stream of nitrogen gas and weighed. For UPLC-PDA-MS^n^, residues were dissolved in ethanol and analyzed by the method of Farrell *et al.* 2017 (ref. 12) following the addition of an equal volume of water. For NMR, residues were dissolved in DMSO-d_6_. For determination of percent yield of reactions, MS parent ion or PDA peak areas at 337 nm were compared among reactant NP and brominated products to determine relative amounts.

For soybean biocatalysis reactions, extracts were analyzed using an Agilent 1290-QTRAP 5500 LC-MS^n^ (AB Sciex Framingham, MA) in precursor ion scanning mode to identify molecules that have lost a bromine during MS^n^ fragmentation. Extract (5 μL) was injected onto a symmetry 3.5 μM, 4.6 × 75 mm C18 column incubated at 30 °C with a flow rate of 1 mL min^−1^. Solvent A and B were acetonitrile and water in 0.1% formic acid, respectively. The composition of A was 20% from 0–2 min, 20–95% from 2–18 min, 95% 18–20 min, then held at 20% for 4 min to stabilize pressure prior to the following injection. MS^n^ settings are listed in ESI, Table S3.†

### Human cell culturing and *in vitro* proliferation assays

HT-29 cells were cultured in DMEM (Gibco, Gaithersburg, MD) with 10% FBS (Fisher Scientific, Hampton, NH). HCT-116 cells were in 45% DMEM, 45% F12, and 10% FBS medium. Cells were cultured in a GALAXY CO48R HT CO_2_ and N_2_-equipped tri-gas incubator (Eppendorf, Hauppauge, NY) at 37 °C, 5% CO_2_, 95% humidity and 19% or 0.5% O_2_ for normoxia and hypoxia, respectively. All compounds were dissolved in DMSO to 10 mM and stored at −80 °C. Treatments were prepared fresh by serially diluting stock solutions to 90, 60, 30, 10, and 5 μM in culture medium. For treatments, trypsinized cells were diluted with culture medium. 5000 cells in 100 μL medium were loaded into a 96-well plate. After 1 hour at 37 °C to allow for cell adhesion, the medium was replaced with 100 μL of medium containing compounds. After 48 hours, 10 μL of MTT reagent (5 mg mL^−1^ in PBS) was added (ThermoFisher, Waltham, MA). After 4 hours, 100 μL of isopropanol/0.04 N HCl was added to dissolve formazan and Abs_570 nm_ was measured. For hypoxia treatments, cells cultured in normoxia were transferred to 0.5% O_2_ for 20 hours then treated with compounds for 24 hours. Results represent an average of the means of two independent experiments, each included three biological replicates, and ±the standard error (S.E.). Data were analyzed using single factor analysis of variance (ANOVA) followed by Tukey's *post hoc* analysis.

## Results

### Biocatalysis and semi-synthesis can be combined to (bio)synthesize 6,8-dibromogenkwanin with different efficiencies depending on their order

To directly test our hypothesis of whether promiscuous NP biosynthesis enzymes can use semi-synthetic NP derivatives as substrates, we first brominated apigenin at 6- and 8-positions using a semi-synthesis protocol that was developed for dihydroquercetin.^9^ We chose to brominate these positions since several flavonoid derivatives that were halogenated at those sites exhibited greater antioxidant activity, increased lipophilicity, and were more effective against human gastric and colorectal adenocarcinoma cell lines compared to their NP precursors.^6,7^

**Fig. 2:**
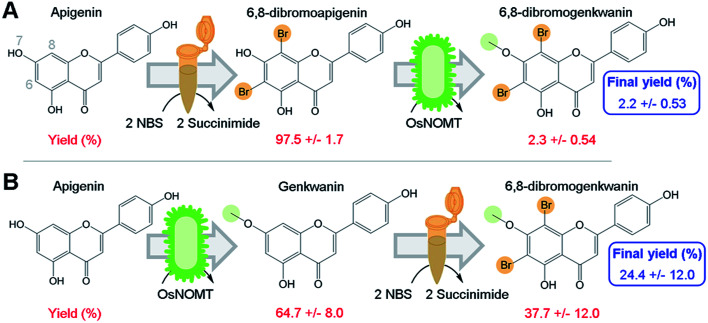
Module order affects the efficiency of (bio)synthesizing 6,8-dibromogenkwanin from apigenin. (A) In reaction scheme 1 apigenin was first converted to 6,8-dibromo-apigenin by semi-synthesis module using the *N*-bromo-succinimide method. 6,8-dibromo-apigenin was then converted to DiBrGenk by the biocatalysis module using bacteria engineered to express the rice enzyme OsNOMT. (B) In reaction scheme 2 the modules were conducted in the reverse order. The percent yields of the individual steps and the cumulative final yield are shown in red and blue fonts, respectively.

Apigenin was converted to 6,8-dibromoapigenin with 97.5% efficiency by semi-synthesis using the bromine donor *N*-bromosuccinimide (Fig. 2A). Reaction products were identified by exact mass LC-PDA-MS^n^ and ^1^H NMR spectroscopy (ESI, Tables S1 and S2†). Purified 6,8-dibromoapigenin was supplied to *E. coli* that was engineered to express a codon-optimized version of the rice (*Oryza sativa*) gene naringenin 7-*O*-methyltransferase from (*OsNOMT*).^13^*E. coli* converted 6,8-dibromoapigenin to 6,8-dibromogenkwanin with 2.3% efficiency (mol mol^−1^), yielding 0.6 mg L^−1^ culture. Thus, the overall efficiency of the tandem (bio)synthesis approach ‘A’ was 2.2% (mol mol^−1^) (Fig. 2A).

We hypothesized that an advantage of conducting semi-synthesis and biocatalysis as separate modules was that the order of the modules could be changed to manipulate reaction efficiency. To test, we did the experiment in the reverse order starting with biocatalysis. OsNOMT-expressing *E. coli* produced genkwanin from apigenin with 64.7% efficiency (mol mol^−1^) yielding 11.0 mg L^−1^ culture. Subsequently, genkwanin was converted to 6,8-dibromogenkwanin with 37.7% efficiency (mol mol^−1^) by semi-synthesis. 6,8-Dibromogenkwanin was the major product, but we also observed lesser amounts of monobrominated and tribrominated derivatives. Thus, the overall efficiency of the tandem (bio)synthesis approach ‘B’ was 24.4% (mol mol^−1^) (Fig. 2B), roughly 11-fold greater than approach ‘A’. The specific activities for apigenin and 6,8-dibromoapigenin substrates were 254.6 and 6.0 fkat mg^−1^ total *E. coli* protein, respectively. However, our metabolite extraction protocol resulted in complete loss of enzyme activity. Overall, our results demonstrated that modules of semi-synthesis and biocatalysis can be used in tandem and in different combinations to manipulate the yield of desired chemical products.

### Dibromogenkwanin has greater antiproliferative activity against colorectal adenocarcinoma cells *in vitro* compared to its natural precursors at low micromolar treatment concentrations

Colorectal cancer is a major cause of morbidity and mortality throughout the world.^14^ It is the third most common cancer and the fourth most common cause of death.^15^ Of the cancers that begin in the colorectal region, the vast majority (over 95%) are classified as adenocarcinomas.^16^

To determine whether 6,8-dibromogenkwanin had enhanced antiproliferative activity compared to its natural precursors genkwanin and apigenin, we compared their antiproliferation activities against colon cancer cells *in vitro*. We chose gland-derived adenocarcinoma HT-29 and lining-derived carcinoma HCT-116 cells because apigenin and genkwanin have well characterized antiproliferative activities against these cell types.^8,17,18^ Bioactivities were tested under normal atmospheric oxygen conditions (normoxia, 19% O_2_) at which apigenin and genkwanin were previously assayed,^8,17,18^ and under hypoxia (0.5% O_2_), a condition that is more physiologically relevant due to its prevalence during tumorigenesis.^19,20^

**Fig. 3:**
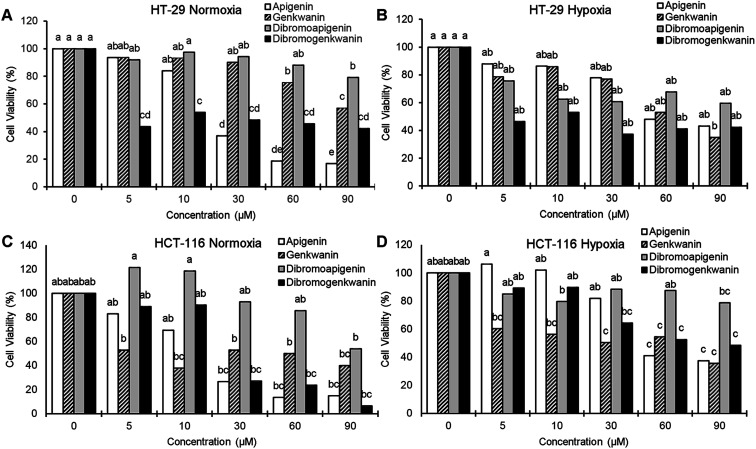
Anti-proliferative effects of natural flavonoids and their 6,8-dibrominated derivatives on HT-29 adenocarcinoma and HCT-116 carcinoma colorectal cells *in vitro*. (A) HT-29 cultured with various concentrations of compound for 48 hours under 19% O_2_. (B) HT-29 pre-cultured for 20 hours under 0.5% O_2_ then cultured with compound under the same condition for 24 hours. (C) HCT-116 cultured for 48 hours under 19% O_2_. (D) HT-29 pre-cultured for 20 hours under 0.5% O_2_ then cultured with compound under the same condition for 24 hours. Different letters denote significant differences, *P* < 0.05, ANOVA, Tukey *post hoc*.

At the lowest treatment concentration compared under normoxia (5 μM), 6,8-dibromogenkwanin inhibited the proliferation of HT-29 adenocarcinoma cells by 56.5%, whereas its NP precursors and 6,8-dibromoapigenin only inhibited proliferation by 6–8% (Fig. 3A). Roughly similar levels of inhibition were observed for the compounds under hypoxia (Fig. 3B). It took 30–60 μM apigenin to cause a similar levels of inhibition. Interestingly, at higher doses, 6,8-dibromogenkwanin did not result in significantly greater levels of inhibition, whereas the levels of inhibition increased for apigenin with increasing concentrations. These results suggested a distinct mechanism of action for 6,8-dibromogenkwanin and apigenin.

In contrast to the effects observed for adenocarcinoma cells, the proliferation of HCT-116 carcinoma cells under normoxia was inhibited equivalently by apigenin and 6,8-dibromogenkwanin (Fig. 3C). The effectiveness of both compounds (and genkwanin) was reduced under hypoxia (Fig. 3D). 6,8-Dibromoapigenin was least effective in both cell types under hypoxia and normoxia.

### Plants for the diversification of NP derivatives: soybean seed coats convert brominated flavonoids into novel derivatives

Various flavonoid biosynthesis enzymes have exhibited promiscuous substrate specificities.^13,21^ As a component of the proposed combinatorial (bio)synthesis approach for generating derivatives of anticancer NPs (Fig. 1), we hypothesized that plant tissues expressing flavonoid enzymes could convert semi-synthetic flavonoids supplied through the culture medium into novel derivatives. Since the semi-synthetic molecule 6,8-dibromoapigenin showed relatively low antiproliferative activities *in vitro*, we decided to test whether soybean seed coats could convert 6,8-dibromoapigenin into novel derivatives. Soybean seed coats from an *I*^*i*^*RT* genotype^11^ were selected since they express genes for flavonoid biosynthesis throughout the seed coat, but lack flavonoids outside the hilum region due to a naturally occurring RNA interference (RNAi) silencing mechanism that silences the expression of the flavonoid biosynthesis genes encoding chalcone synthase (CHS) in those tissues.^22^ Thus *I*^*i*^*RT* genotypes have a yellow seed coat devoid of flavonoids but with all the enzymatic capacity to biosynthesize, modify, and polymerize flavonoid intermediates.

**Fig. 4:**
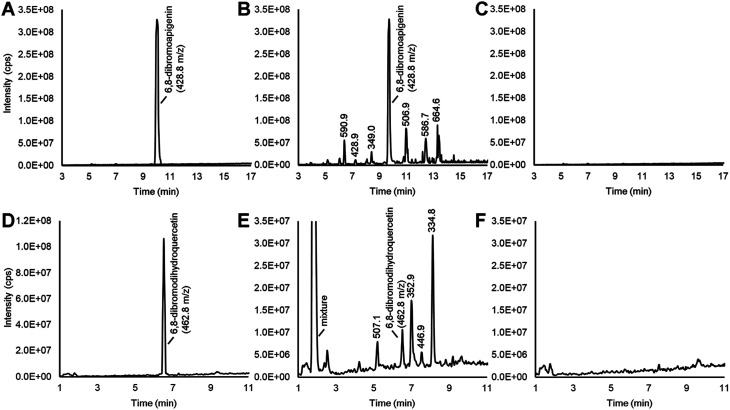
LC-MS^n^ chromatograms in precursor ion measuring derivatives of brominated flavonoids that were produced by soybean seed coats. (A) 6,8-Dibromoapigenin. (B) Brominated molecules that arose from culturing soybean seed coats with 6,8-dibromoapigenin. (C) No brominated molecules were detected after culturing soybean seed coats with apigenin. (D) 6,8-Dibromodihydroquercetin. (E) Brominated molecules that arose from culturing soybean seed coats with 6,8-dibromodihydroquercetin. (F) No brominated molecules were detected after culturing soybean seed coats with dihydroquercetin.

Dibromoapigenin supplied through the culture medium was converted to more than six brominated derivatives (Fig. 4A and B). The parent masses of the brominated metabolites were identified by an LC-MS^n^ method operating in precursor ion mode that specifically identifies molecules that have undergone the loss of bromine groups during MS^n^ fragmentation of parent molecules (see Methods). To ensure that the detected metabolites were specifically derivatives of 6,8-dibromoapigenin, we supplied seed coats apigenin as a comparator. No brominated molecules were detected from seed coats that were supplied apigenin (Fig. 4C).

Soybeans do not biosynthesize apigenin or its derivatives.^11^ To determine whether seed coats could metabolize a brominated version of a native flavonoid intermediate, we fed seed coats 6,8-dibromodihydroquercetin. Dihydroquercetin is an intermediate in the biosynthesis of anthocyanins and proanthocyanidin (PA, a.k.a. condensed tannin) flavonoids. The vast majority was converted to a mixture of highly polar brominated molecules, yet distinct brominated metabolites with greater and lower molecular masses than 6,8-dibromodihydroquercetin were also detected (Fig. 4D and E). Again, no brominated metabolites were measured from seed coats that were supplied the non-brominated flavonoid precursor (Fig. 4F).

## Discussion

Here, we demonstrated a novel approach for making a desired derivative of an ACNP that could not be produced using either semi-synthesis or biocatalysis protocols alone. By combining modules of semi-synthesis and biocatalysis in tandem and in different combinations, we were able to use a novel combined method, named the combinatorial (bio)synthesis approach, to make 6,8-dibromogenkwanin with varying efficiency depending on the module order. Brominated and methylated derivatives of apigenin were previously semi-synthesized to test for enhanced antiproliferative activity against HepG2 hepatoma cells.^23^ However, the approach could not semi-synthesize 6,8-dibromogenkwanin since the methylation of the 7-hydroxyl of apigenin was not a favorable reaction. Biocatalysis is typically an option for producing compounds that are difficult to semi-synthesize. Enzymes that catalyze the halogenation of NPs have been identified mainly from bacteria, but also from plants.^24^ Chloroperoxidase enzymes from *Caldariomyces fumago* were capable of halogenating the flavanones, naringenin and hesperetin, at C-6 and C-8 in the presence of either Cl^−^ or Br^−^. However, they did not accept flavones as substrates.^25^ Here, we demonstrated that the enzyme naringenin 7-*O*-methyltransferase from rice (OsNOMT) expressed in *E. coli* could methylate the 7-hydroxyl of 6,8-dibromoapigenin. Yet, bioconversion to 6,8-dibromogenkwanin occurred with only 2.3% efficiency. Yet, if the module order was reversed and OsNOMT was provided apigenin as a substrate, genkwanin could be biosynthesized with 64.7% efficiency. Genkwanin could then be dibrominated by semi-synthesis using NBS to yield 6,8-dibromogenkwanin with up to 49.7% efficiency. Random mutagenesis screens could potentially be used to improve the catalytic efficiency of OsNOMT towards apigenin or 6,8-dibromoapigenin. For example, this approach generated variants of human theta class 1-1 glutathione transferase enzyme (hGSTT1-1) that exhibited up to a 20 000-fold increased *k*_cat_/*K*_M_ for a chlorinated derivative of coumarin.^26^ Random mutagenesis can also be used to expand the promiscuity of enzymes,^27^ thus further enhancing their usefulness as tools in the combinatorial (bio)synthesis toolbox.

Combinatorial (bio)synthesis relies heavily on the promiscuity of enzymes. Here, we demonstrated that plant tissues should be considered for use as modules since soybean seed coats could readily convert brominated flavonoids fed through the culture medium into novel brominated derivatives. While the identities of the novel derivatives and their activities against cancer would require much more extensive characterization, in this work we have proven the concept that plant tissues can be used as biocatalyst, an area of research that deserves more investigation in the future. Many, or most plant secondary metabolism enzymes are thought to be promiscuous, accepting multiple substrates. This is thought to have been a major contributor to the evolution of chemodiversity in plants.^28,29^ Yet, the promiscuity of enzymes remains poorly understood, and thus cannot be readily predicted based on amino acid sequence or even protein modeling.^26–28^ This can limit our ability to predict whether the desired products will be biosynthesized from an exogenous input molecule. For instance, transgenic roots of the medicinal plant *Catharanthus roseus* (Madagascar periwinkle) engineered to express tryptophan halogenating enzymes from a soil bacterium were found to biosynthesize chlorinated alkaloid intermediates but not the anticancer final biosynthetic products vincristine and vinblastine.^30^ The accumulation of intermediates could be helpful in identifying gene targets for expanding promiscuity by random mutagenesis,^27^ suggesting a focus for future research.

6,8-dibromogenkwanin was made using the combinatorial (bio)synthesis approach and had enhanced antiproliferative activity against HT-29 colorectal adenocarcinoma cells compared to its natural precursors apigenin and genkwanin at low (5 μM) treatment concentrations. As a preliminary glimpse into its mechanism of action, its inhibition of proliferation of HT-29 cells seemed to plateau at higher treatment concentrations whereas apigenin increased at higher concentrations. These results may suggest at least a partially distinct mechanism of 6,8-dibromogenkwanin and its natural counterpart. Future research should investigate more intensively the mechanism of action of this molecule and determine its efficacy for treating adenocarcinoma tumors in animal models.

## Conclusions

In summary, the combinatorial (bio)synthesis approach combines modules of semi-synthesis and biocatalysis to produce novel derivatives of NPs that could not be obtained using the current protocols of either method alone. This opens a new avenue for assembling novel chemistries for the fight against cancer.

## Conflicts of interest

The authors declare no financial or commercial conflict of interest.

## Supplementary Material

RA-008-C8RA02184H-s001
