# Supplementary material for: Combining semi-synthesis with plant and microbial biocatalysis: new frontiers in producing a chemical arsenal against cancer
Source: RSC Adv. 2018 Jun 11;8(38):21332–9. doi: 10.1039/c8ra02184h (PMC9080863; doi:10.1039/c8ra02184h)
Supplement: RA-008-C8RA02184H-s001 [file RA-008-C8RA02184H-s001.pdf]

Electronic Supplementary Material (ESI) for RSC Advances.
This journal is © The Royal Society of Chemistry 2018

Table S1. UPLC-PDA-MS^n identifying features of bromination reaction products and substrates.

Peak	R_t (min)	λ_{max} (nm)	$[M - H]^-$	$[M + H]^+$	MS/MS fragments$^+$ (m/z)
apigenin[a]	10.75	218, 267s, 335s	269.05	271.05	117.03
genkwanin[a]	13.26	221, 267s, 334s	283.06	285.05	117.03
6,8-dibromogenkwanin[b]	17.52	222, 275s, 332s	440.88	442.88	425.86
tribromo-genkwanin	18.00	223, 272s, 331s	518.79	520.79	299.20
bromo-genkwanin A	15.28	220, 272s, 333s	360.97	362.97	-
bromo-genkwanin B	16.12	221, 271s, 331s	360.97	362.97	-
6,8-dibromoapigenin[b]	16.11	221, 276s, 332s	426.86	428.86	-

[a]Peak identity based on comparison to an authentic standard.

[b]Identity based on exact mass and proton NMR.

Table S2. 1H Chemical Shifts (ppm) of Brominated Flavone Derivatives.

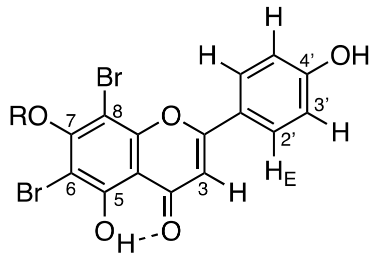

	3-H	5-OH	7-OR	4-OH	2'-H	3'-H	$J_{2'3'}$ [c]
6,8-dibromo-apigenin (R = H)	6.73	13.80[a]	10.97[a]	10.97[a]	7.85	6.87	8.8
6,8-dibromo-genkwanin (R = CH_3)	7.16	13.98[c]	3.97	10.59[c]	8.09	7.02	8.8

Solvent DMSO-d_6: a) broad singlet b) broad singlet; c) sharp singlet

Table S3. Agilent 1290-QTRAP 5500 LC-MS^n Settings

Quantitation Table:

Period 1:

Scans in Period:3395
Relative Start Time: 60000.00 msec
Experiments in Period: 5

IDA Properties:

First Criteria:
Intense peaks from 1 to 3
Ions greater than: 260.000 m/z
Ions smaller than: 730.000 m/z
Which exceeds 5000.000 counts
Rolling collision energy: No
Exclude former target ions: Never
Mass Tolerance: 250.000 mDa
Exclude isotopes within 4.0 Da window
Use Enhanced Resolution scan to confirm Charge State and\or Isotope Pattern selection: Yes
Exclude 1+ precursors from Enhanced Resolution Scan confirmation and MS/MS:Yes
Dynamic Background Subtraction:Yes

Period 1 Experiment 1:

Scan Type: Precursor Ion (Prec)
Polarity: Negative
Scan Mode: Profile
Ion Source: Turbo Spray
Scans to Sum:1
Precursor Of: 80.90 Da
Resolution Q1: Unit
Resolution Q3: Unit
Scan Rate: 1000 Da/s
Intensity Thres.: 0.00 cps
Settling Time: 0.0000 msec
MR Pause: 5.0070 msec
MCA: No
Center/Width: No
Step Size: 0.10 Da

Start (Da)	Stop (Da)	Time (sec)	Param	Start	Stop
260.00	730.00	0.47			

Parameter Table(Period 1 Experiment 1)
CUR: 35.00
IS: -4500.00
TEM: 650.00

GS1: 60.00
GS2: 60.00
CAD: High
DP -100.00
EP -10.00
CE -100.00
CXP -15.00

Period 1 Experiment 2:

Scan Type: Enhanced Resolution (ER)
Polarity: Negative
Scan Mode: Profile
Ion Source: Turbo Spray
Scans to Sum: 1
Resolution Q1: Open
Scan Rate: 250 Da/s
Intensity Thres.: 0.00 cps
Settling Time: 0.0000 msec
MR Pause: 15.0000 msec
Q0 trapping: No
MCA: No
Center/Width: Yes
LIT fill time: 0.05 msec
Dynamic Fill Time: On

TIC Target	EMS Scan:	10.00 x1e7 cps.
TIC Target	ER Scan:	1.00 x1e7 cps.
TIC Target	EPI Scan:	10.00 x1e7 cps.
Max Fill	EMS Scan:	150.000 msec
Max Fill	ER Scan:	250.000 msec
Max Fill	EPI Scan:	250.000 msec
Min Fill	EMS Scan:	0.050 msec
Min Fill	ER Scan:	0.050 msec
Min Fill	EPI Scan:	0.050 msec
Default Fill	EMS Scan:	0.050 msec
Default Fill	ER Scan:	0.050 msec
Default Fill	EPI Scan:	1.000 msec

Q3 Entry Barrier: 8.00 V
Step Size: 0.02 Da

Start (Da)	Stop (Da)	Time (sec)	Param	Start	Stop
<Best ion>		AF3	0.08	0.08	
	EXB	158.78	158.72		

Start (Da)	Stop (Da)	Time (sec)	Param	Start	Stop
<Best ion>		AF3	0.08	0.08	
	EXB	158.78	158.72		

Start (Da)	Stop (Da)	Time (sec)	Param	Start	Stop
<Best ion>		AF3	0.08	0.08	
	EXB	158.78	158.72		

Parameter Table(Period 1 Experiment 2)

CUR:	35.00
IS:	-4500.00
TEM:	650.00
GS1:	60.00
GS2:	60.00
CAD:	High
DP	-100.00
EP	-10.00
CE	-10.00
CES	-0.00

Period 1 Experiment 3:

Scan Type:	Enhanced Product Ion (EPI)	
Polarity:	Negative	
Scan Mode:	Profile	
Ion Source:	Turbo Spray	
# Scans to Sum:	1	
Product Of:	30.00 Da	
Resolution Q1:	Unit	
Scan Rate:	10000 Da/s	
Intensity Thres.:	0.00 cps	
Settling Time:	0.0000 msec	
MR Pause:	1.5000 msec	
Q0 trapping:	No	
MCA: No		
Center/Width:	No	
LIT fill time:	1.00 msec	
Dynamic Fill Time:	On	
TIC Target	EMS Scan:	10.00 x1e7 cps.
TIC Target	ER Scan:	1.00 x1e7 cps.
TIC Target	EPI Scan:	10.00 x1e7 cps.
Max Fill	EMS Scan:	150.000 msec
Max Fill	ER Scan:	250.000 msec
Max Fill	EPI Scan:	250.000 msec
Min Fill	EMS Scan:	0.050 msec
Min Fill	ER Scan:	0.050 msec
Min Fill	EPI Scan:	0.050 msec
Default Fill	EMS Scan:	0.050 msec
Default Fill	ER Scan:	0.050 msec
Default Fill	EPI Scan:	1.000 msec

Q3 Entry Barrier: 8.00 V
Step Size: 0.12 Da

Start (Da)	Stop (Da)	Time (sec)	Param	Start	Stop
50.00	102.87	0.0053	AF3	2.98	3.06
			EXB	155.79	153.89

Start (Da)	Stop (Da)	Time (sec)	Param	Start	Stop
102.87	308.63	0.0206	AF3	3.06	3.40
			EXB	153.89	146.52

Start (Da)	Stop (Da)	Time (sec)	Param	Start	Stop
308.63	730.00	0.0421	AF3	3.40	4.10
			EXB	146.52	131.41

Parameter Table(Period 1 Experiment 3)
CUR: 35.00
IS: -4500.00
TEM: 650.00
GS1: 60.00
GS2: 60.00
CAD: High
DP -100.00
EP -10.00
CE -100.00
CES 40.00

Period 1 Experiment 4:

Scan Type: Enhanced Product Ion (EPI)
Polarity: Negative
Scan Mode: Profile
Ion Source: Turbo Spray
Scans to Sum: 1
Product Of: 30.00 Da
Resolution Q1: Unit
Scan Rate: 10000 Da/s
Intensity Thres.: 0.00 cps
Settling Time: 0.0000 msec
MR Pause: 1.5000 msec
Q0 trapping: No
MCA: No
Center/Width: No
LIT fill time: 1.00 msec
Dynamic Fill Time: On

TIC Target	EMS Scan:	10.00 x1e7 cps.
TIC Target	ER Scan:	1.00 x1e7 cps.
TIC Target	EPI Scan:	10.00 x1e7 cps.
Max Fill	EMS Scan:	150.000 msec
Max Fill	ER Scan:	250.000 msec
Max Fill	EPI Scan:	250.000 msec
Min Fill	EMS Scan:	0.050 msec
Min Fill	ER Scan:	0.050 msec
Min Fill	EPI Scan:	0.050 msec
Default Fill	EMS Scan:	0.050 msec
Default Fill	ER Scan:	0.050 msec
Default Fill	EPI Scan:	1.000 msec

Q3 Entry Barrier: 8.00 V
Step Size: 0.12 Da

Start (Da)	Stop (Da)	Time (sec)	Param	Start	Stop
50.00	102.87	0.0053	AF3	2.98	3.06
			EXB	155.79	153.89

Start (Da)	Stop (Da)	Time (sec)	Param	Start	Stop
102.87	308.63	0.0206	AF3	3.06	3.40
			EXB	153.89	146.52

Start (Da)	Stop (Da)	Time (sec)	Param	Start	Stop
308.63	730.00	0.0421	AF3	3.40	4.10
			EXB	146.52	131.41

Parameter Table(Period 1 Experiment 4)

CUR:	35.00
IS:	-4500.00
TEM:	650.00
GS1:	60.00
GS2:	60.00
CAD:	High
DP	-100.00
EP	-10.00
CE	-100.00
CES	40.00

Period 1 Experiment 5:

Scan Type:	Enhanced Product Ion (EPI)
Polarity:	Negative
Scan Mode:	Profile
Ion Source:	Turbo Spray

Scans to Sum: 1
Product Of: 30.00 Da
Resolution Q1: Unit
Scan Rate: 10000 Da/s
Intensity Thres.: 0.00 cps
Settling Time: 0.0000 msec
MR Pause: 1.5000 msec
Q0 trapping: No
MCA: No
Center/Width: No
LIT fill time: 1.00 msec
Dynamic Fill Time: On

TIC Target	EMS Scan:	10.00 x1e7 cps.
TIC Target	ER Scan:	1.00 x1e7 cps.
TIC Target	EPI Scan:	10.00 x1e7 cps.
Max Fill	EMS Scan:	150.000 msec
Max Fill	ER Scan:	250.000 msec
Max Fill	EPI Scan:	250.000 msec
Min Fill	EMS Scan:	0.050 msec
Min Fill	ER Scan:	0.050 msec
Min Fill	EPI Scan:	0.050 msec
Default Fill	EMS Scan:	0.050 msec
Default Fill	ER Scan:	0.050 msec
Default Fill	EPI Scan:	1.000 msec

Q3 Entry Barrier: 8.00 V
Step Size: 0.12 Da

Start (Da)	Stop (Da)	Time (sec)	Param	Start	Stop
50.00	102.87	0.0053	AF3	2.98	3.06
			EXB	155.79	153.89

Start (Da)	Stop (Da)	Time (sec)	Param	Start	Stop
102.87	308.63	0.0206	AF3	3.06	3.40
			EXB	153.89	146.52

Start (Da)	Stop (Da)	Time (sec)	Param	Start	Stop
308.63	730.00	0.0421	AF3	3.40	4.10
			EXB	146.52	131.41

Parameter Table(Period 1 Experiment 5)
CUR: 35.00
IS: -4500.00
TEM: 650.00
GS1: 60.00
GS2: 60.00
CAD: High
DP -100.00

EP	-10.00
CE	-100.00
CES	40.00

Resolution tables

Quad 1 Negative Unit Scan Speed = 1000 Da/s

Last Modification Date Time: June 20, 2012 12:07:02

IE1 -0.500

Mass (Da)	Offset Value
44.998	-0.042
411.259	0.119
585.385	0.200
933.636	0.315

Quad 3 Negative Low Scan Speed = 10 Da/s

Last Modification Date Time: August 13, 2013 09:56:00

Mass (Da)	Offset Value
44.998	-0.450
411.259	-1.160
585.385	-1.515
933.636	-2.212

Quad 3 Negative Unit Scan Speed = 10 Da/s

Last Modification Date Time: December 27, 2012 14:14:08

IE3 0.000

Mass (Da)	Offset Value
44.998	-0.150
411.259	-0.860
585.385	-1.215
933.636	-1.912

Calibration tables

Quad 1 Negative Unit Resolution Scan Speed = 10 Da/s

Last Modification Date Time: December 28, 2012 10:35:40

Mass (Da)	Dac Value
44.998	8087
585.385	106766
933.636	170369

Quad 1 Negative Unit Resolution Scan Speed = 1000 Da/s

Last Modification Date Time: December 28, 2012 10:46:48

Mass (Da)	Dac Value
44.998	8115
411.259	75034
585.385	106853
933.636	170471

Quad 3 Negative Unit Resolution Scan Speed = 10 Da/s
Last Modification Date Time: December 27, 2012 14:14:34

Mass (Da)	Dac Value
44.998	8183
411.259	75808
585.385	107961
933.636	172261

Quad 3 Negative Unit Resolution Scan Speed = 2000 Da/s
Last Modification Date Time: June 20, 2012 12:19:58

Mass (Da)	Dac Value
44.998	8222
411.259	75883
585.385	108047
933.636	172388

Quad 3 Negative Scan Speed = 250 Da/s
Last Modification Date Time: June 20, 2012 14:59:12

Mass (Da)	Dac Value
112.985	24976
431.982	95599
601.978	133225

Quad 3 Negative Scan Speed = 10000 Da/s
Last Modification Date Time: June 20, 2012 15:36:46

Mass (Da)	Dac Value
112.985	25058
431.982	95853
601.978	133532

Instrument Parameters:
Detector Parameters (Negative):
CEM 2200.0
